# Identifying effective candidates for sacro-iliac joint fusion

**DOI:** 10.2340/17453674.2024.41305

**Published:** 2024-08-21

**Authors:** Daisuke Kurosawa, Bengt Sturesson

**Affiliations:** Department of Orthopaedic Surgery/Japan Sacroiliac Joint and Low Back Pain Center, JCHO Sendai Hospital, Sendai, Japan; Aleris specilistvård, Ängelholm, Sweden

*Sir,*–We appreciated reading “Patient-reported outcomes after minimally invasive sacro-iliac joint (SIJ) surgery: a cohort study based on the Swedish Spine Registry” by Randers et al. [1]. Their paper presents a thoughtful analysis, suggesting that SIJ fusion may not be as effective as previously considered, aligning with their earlier randomized-controlled trial (RCT) comparing SIJ fusion with sham surgery [2].

In practice, however, we often see patients improve significantly after SIJ fusion, resuming normal activities, especially those who did not benefit from lumbar surgeries. A previous study shows that 78% of patients had good outcomes from SIJ fusion [3], which is generally similar to studies using conventional surgical techniques. Industry-sponsored studies may highlight the effectiveness of minimally invasive SIJ fusion [4]. However, our clinical experience suggests a real benefit to SIJ fusion.

The study reported only a modest numerical rating scale (NRS) improvement from a preoperative average of 6.7 to 4.4 at 2 years postoperatively. This average likely includes some well-improved cases but is influenced by the fact that 87% of the surgical cases were women. Women tend to be more vulnerable to pelvic problems due to structural differences in the SIJ that allow for greater joint motion. Therefore, fusion may place additional strain on surrounding areas such as the contralateral SIJ, pubic symphysis, L5–S1 junction, and iliolumbar ligament, which may explain some of the observed differences in pain and functional outcomes.

The inclusion criteria for diagnosing SIJ dysfunction in their previous RCT study [2] seem ideal, and similar criteria may be used by surgeons in Sweden/Norway. However, conventional diagnostics may miss severe cases requiring surgery. The most important confounding factor is etiology, where post-traumatic patients have the best prognosis in our experience. The primary indication for fusion surgery is the chronic inability of the SIJ to function as a load-bearing joint. This conclusion is based on observing changes in load bearing after pelvic external fixation [5].

The other confounding factors is the diagnostic procedure and recruitment of patients. SIJ fusion surgery was not registered in Swespine before 2013 or 2014. During 2013 to 2020, SIJ fusions were performed in 3 centers in Sweden. The diagnostic procedure in Ängelholm included intra-articular SIJ injections, radiographically confirmed with contrast medium. Whether or not other centers diagnosed the patient with the same cautious SIJ injections is uncertain. As surgeries were performed at 3 different centers, it would be valuable to compare their outcomes. Unfortunately, there is considerable missing information from the hospital in Ängelholm. During this period there were several administrative changes affecting the registration of the preoperative patient-reported outcome measures (PROMs), markedly reducing the number of patients in the study.

According to the findings of Randers et al., surgeons should carefully assess the criteria for surgical intervention in severe SIJ dysfunction cases that are unresponsive to conservative treatments. Evaluating the potential benefits of surgery using pelvic external fixation may be advisable. Further research is needed to refine these criteria and ensure accurate identification of patients who would benefit most from surgery.
